# Gomisin M2 from Baizuan suppresses breast cancer stem cell proliferation in a zebrafish xenograft model

**DOI:** 10.18632/aging.102323

**Published:** 2019-10-14

**Authors:** Yeguo Yang, Erwei Hao, Xianglong Pan, Dechao Tan, Zhengcai Du, Jinling Xie, Xiaotao Hou, Jiagang Deng, Kun Wei

**Affiliations:** 1 School of Biology and Biological Engineering, South China University of Technology, Guangzhou 510006, China; 2 Guangxi Key Laboratory of Efficacy Study on Chinese Materia Medica, Guangxi University of Chinese Medicine, Nanning, Guangxi 530200, China; 3 Sino-Canada Joint Zebrafish Lab for Chinese Herbal Drug Screening, Guangxi University of Chinese Medicine, Nanning, Guangxi 530200, China; 4 Guangxi Collaborative Innovation Center for Research on Functional Ingredients of Agricultural Residues, Guangxi University of Chinese Medicine, Nanning, Guangxi 530200, China

**Keywords:** Gomisin M2, breast cancer stem cell, zebrafish xenografts, proliferation

## Abstract

Gomisin M2 isolated from Schisandra viridis A. C. Smith has potential anti-tumor effects on certain cancers, including breast cancer. However, only a few investigations have been conducted on the effects of Gomisin M2 on breast cancer stem cells (CSCs), which have the ability to self-renew and differentiate, as a possible strategy to resolve cancer cell resistance to apoptosis and to improve treatments. It is essential to investigate the effects of Gomisin M2 on breast cancer stem cells (BCSCs). In this study, we enriched breast cancer stem cells with CD44+/CD24- from MDA-MB-231 and HCC1806 cells through magnetic-activated cell sorting and cultured these in serum-free medium. The ability of Gomisin M2 to kill breast cancer stem cells was evaluated *in vitro* and *in vivo*. Gomisin M2 significantly inhibited the proliferation of the triple-negative breast cancer cell lines and mammosphere formation in breast CSCs and downregulated the Wnt/β-catenin self-renewal pathway. Moreover, Gomisin M2 induced apoptosis and blocked the mitochondrial membrane potential of BCSCs. Gomisin M2 suppressed the proliferation of MDA-MB-231 and HCC1806 xenografts in zebrafish. Together, these findings suggest that the anti-BCSC activity of Gomisin M2 could become a promising starting point for the discovery of novel BCSC-targeting drugs.

## INTRODUCTION

Breast cancer is one of the most common female malignancies worldwide, and its incidence continues to increase [1]. In the United States, it has been estimated that approximately 268,600 new breast cancer cases and 41,760 deaths would occur in 2019 [2]. Even in developing countries such as China, breast cancer ranks as the sixth common cause of death among females [3]. Triple-negative breast cancer (TNBC) is a special breast cancer subtype that is difficult to treat [4]. The lack of effective targeted therapies for TNBC is closely related to chemoresistance, higher rates of cell metastasis, and enriched cancer stem cell (CSC) populations in tumors [5, 6]. Emerging evidence suggests that cancer stem cells (CSCs) play a crucial role in breast cancer metastasis, recurrence, and drug resistance. However, current interventions for cancer stem cells are in the pre-clinical research stage, so there is no mature method for targeting tumor stem cell function. In recent years, there has been convincing evidence that Chinese medicines (CMs) and their natural active compounds can be involved in the regulation of stem cell properties. A multitude of CMs and their natural active compounds such as Antrodia camphorate [7], berberine [8, 9], resveratrol [10–12], curcumin [13–18] and sulforaphane [19–21] have been demonstrated to regress CSCs. Therefore, it is important to find more effective natural compounds to target CSCs, especially to cure breast cancer.

Ethnic Yao medicine is a special branch of traditional Chinese medicine. Zuan is one of the species of medicinal plants that has been used as Yao ethnomedicine. There is growing evidence indicating that the Zuan groups are Yao medicines that have anti-tumor effects. Wang et al. extracted four acids from Dazuan that can significantly inhibit the proliferation of human leukemia HL-60 cells [22]. Shuanggou Zuan has been shown to inhibit the proliferation of multiple cancer cell lines, including HCT-15, A549, HT-1197, and MCF-7 cell lines *in vitro* [23]. Hou et al. confirmed that Baizuan has a certain inhibitory effect on MCF7 and CAL27 cell activity and isolated and identified six compounds with antitumor activity [24].

Gomisin M2 is a natural product extracted from Baizuan (ethnic Chinese Yao medicine) that is used as an anti-cancer medicine. In this study, we screened the best-performing compound Gomisin M2 extracted from Baizuan in MDA-MB-231 and HCC1806 breast cancer cell lines. Although it has been reported that Gomisin M2 inhibits breast cancer cell proliferation [24], the molecular mechanism and function of this compound in BCSCs have not been elucidated. Based on these data, we can infer that Gomisin M2 has potent anticancer activity in breast cancer cell lines and breast CSCs zebrafish xenograft model *in vivo*. Interestingly, we found that Gomisin M2 inhibited the signaling pathway of Wnt/β-catenin. These findings suggest that Gomisin M2 may be potentially used as a therapeutic agent in targeting BCSCs and warrants further investigation.

## RESULTS

### Gomisin M2 inhibits TNBC cell line viability in a dose-dependent manner and decreases the size of three-dimensional (3D) spheroid formation in a time-dependent manner

**Figure 1:**
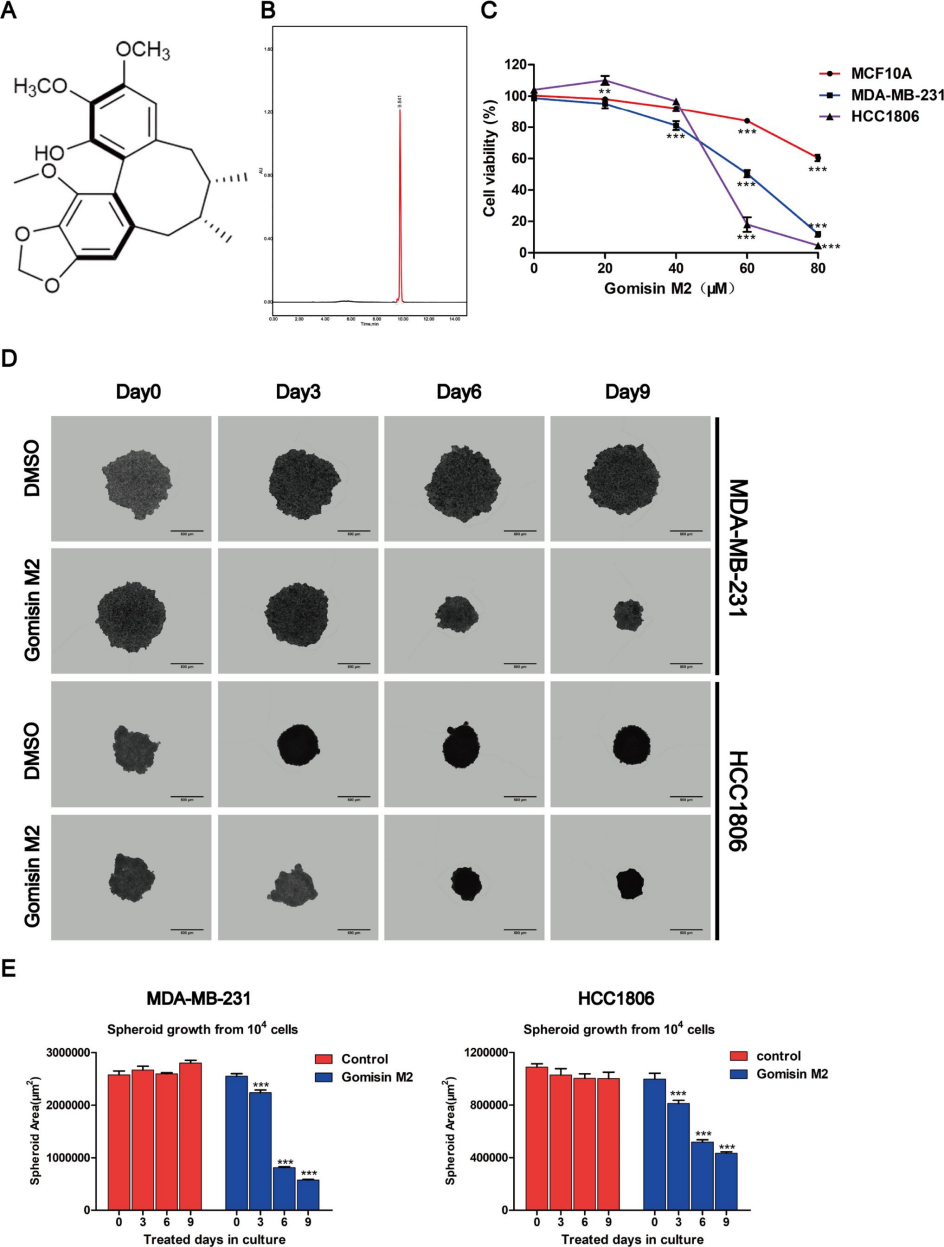
**Effects of Gomisin M2 on the viability of MCF10A, MDA-MB-231, and HCC1806 cells.** (**A**) The chemical structure of Gomisin M2. (**B**) The HPLC chromatograms of Gomisin M2. (**C**) Cells were treated with increasing doses of Gomisin M2 for 48 h. Cell viability determined by Alamar blue assay. (**D**) Images of the 3D spheroids that were treated with Gomisin M2 over 9 days were acquired in all microplates using the PerkinElmer Operetta High-Content Imaging System. Scale bar = 200 μm. (**E**) Bar plot of the average cross-sectional area of the MDA-MB-231 and HCC1806 spheroids. Approximately three replicate tumor spheroid samples were used for quantification. The data were expressed as the mean ± SD. Compared with the DMSO group: **p < 0.01.

To investigate the anti-cancerous compound Gomisin M2 (Figure 1A). extracted from Baizuan that has been reported in previous studies [24], two human TNBC cell lines, namely, MDA-MB-231 and HCC1806, were treated with Gomisin M2 for 2 days, and their cell viability was determined using the Alamar blue assay. The immortalized breast epithelial cell line MCF10A was used as controls. We found that the Gomisin M2 inhibited the proliferation of two TNBC cell lines in a dose-dependent manner. The IC_50_ of Gomisin M2 after 48 h of treatment was 85, 60, 57 μM in three cell lines (MCF10A, MDA-MB-231 and HCC1806) respectively. However, the viability of normal breast epithelial cell line MCF10A was not inhibited by Gomisin M2, and IC50 values were > 80 μM (Figure 1C). Cells subjected to 3D culture can better simulate natural cellular interactions and mimic *in vivo* microarchitecture. In many systems, 3D cell culture methods can offer a more physiologically relevant context over traditional cell culture models for the screening and identification of active compounds. The MDA-MB-231 and HCC1806 cells were seeded into ULA 96-well flat bottom plates at a density of 10,000 cells/well. The cells were exposed to Gomisin M2 at a concentration of 100 μM and allowed to grow for nine days to form spheroids. We assessed the size of the spheroids in relation to time in culture (Figure 1D). Spheroid size significantly decreased after Gomisin M2 treatment for over 9 days in culture. The cross-sectional spheroid area was measured with Harmony software of a high-content imaging system (Figure 1E).

### Identification of BCSC markers in normal breast cancer cell lines

**Figure 2:**
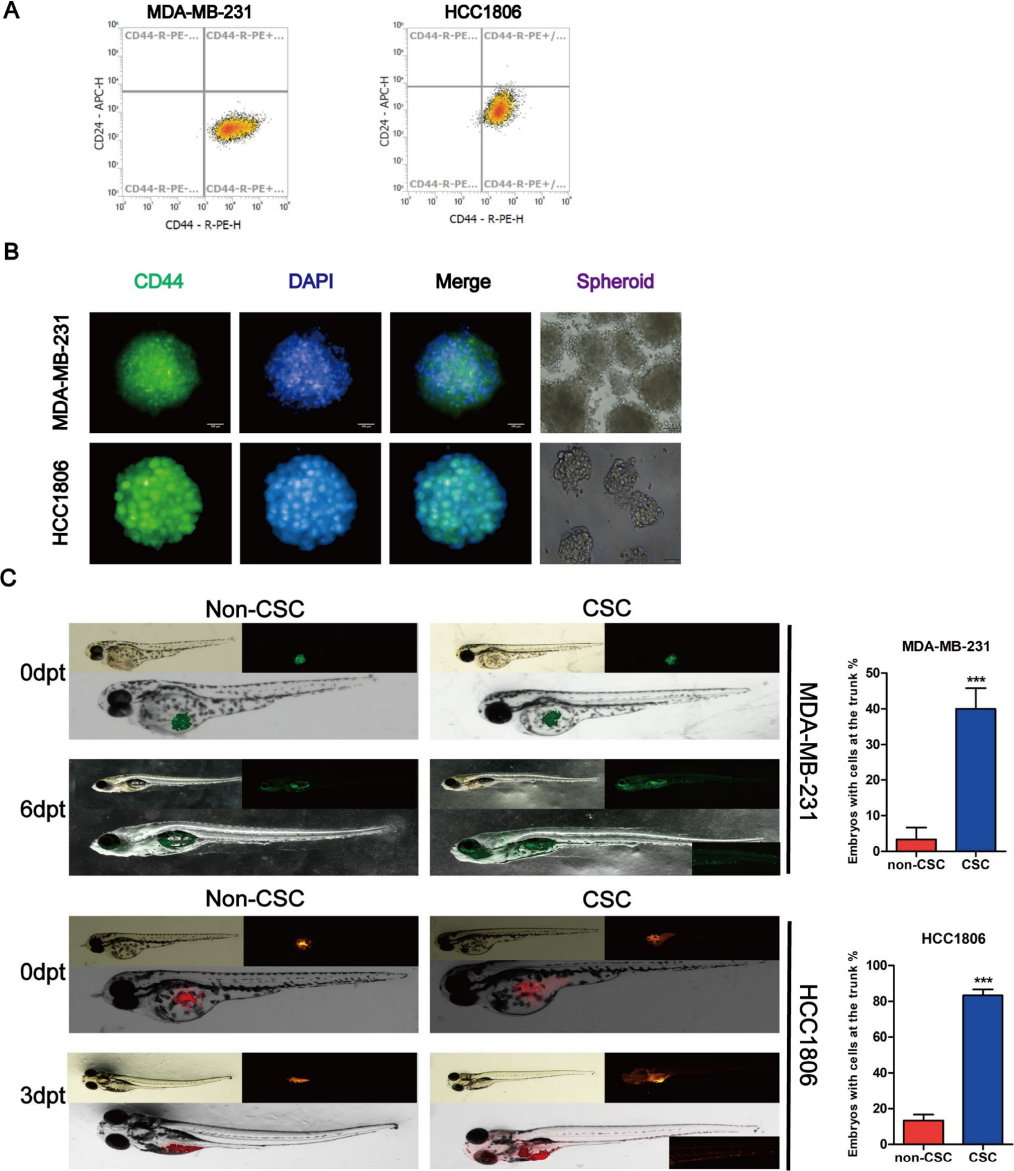
**Identification of stem cell-like properties in breast cancer cell lines.** (**A**) Identification of the purity of tumor stem cell markers sorted from magnetic beads. (**B**) MDA-MB-231 and HCC1806 tumor spheres were dyed with antibodies for breast cancer stem cell-related marker and DAPI for nuclei. Fluorescence images were captured using an Operetta® High Contents Screening System. (**C**) A representative image of MDA-MB-231-GFP (green) and DiI-labeled HCC1806 (red) cells that were implanted in zebrafish embryos at 48 hpf. Non-CSC cells remained in the yolk sac, whereas CSC cells were distributed to the tail (lower right part shows the magnification image of cell migration (larvae stage, n = 30 per group). Right panel, analysis of the percentage of fish with cells at the trunk.

Prior investigations of BCSCs have been conducted using cancer cell lines or patient primary tumor tissue samples, of which, the former is more often used due to easier access. In this study, we sorted cancer stem cells according to the marker of BCSCs by magnetic-activated cell sorting (MACS). We isolated CD44+/CD24- cells from the normal cancer cells with MACS and detected CD44 and CD24 expression to determine CD44 purity by flow cytometry. Cytometry analysis of the proportion of cancer stem cells (CD44+/CD24-) isolated with MACS was > 99% (Figure 2A). We found that the BCSCs had the ability form tumor spheres, and CD44 significantly increased in tumor spheres using a high-content system immunofluorescence (Figure 2B). A small population of cells that were CD44+/CD24- formed tumor spheres. We transplanted 200–300 cancer stem cells harvested from tumor spheres and non-cancer stem cells and injected these into 2 days post-fertilization (dpf) zebrafish embryos to assess their proliferation and migratory behaviour. MDA-MB-231-GFP cells derived from mammospheres in 2-dpf zebrafish embryos were observed to migrate to the trunk on day 6 after cell transplantation. Moreover, the number of fluorescent particles increased compared to the non-CSC group in the zebrafish xenograft. However, the HCC1806 cells labeled with DiI and derived from mammospheres were migrated to the trunk of 2-dpf zebrafish embryos on day 3 post cell transplantation (Figure 2C).

### Gomisin M2 inhibits breast cancer stem cells *in vitro*

**Figure 3:**
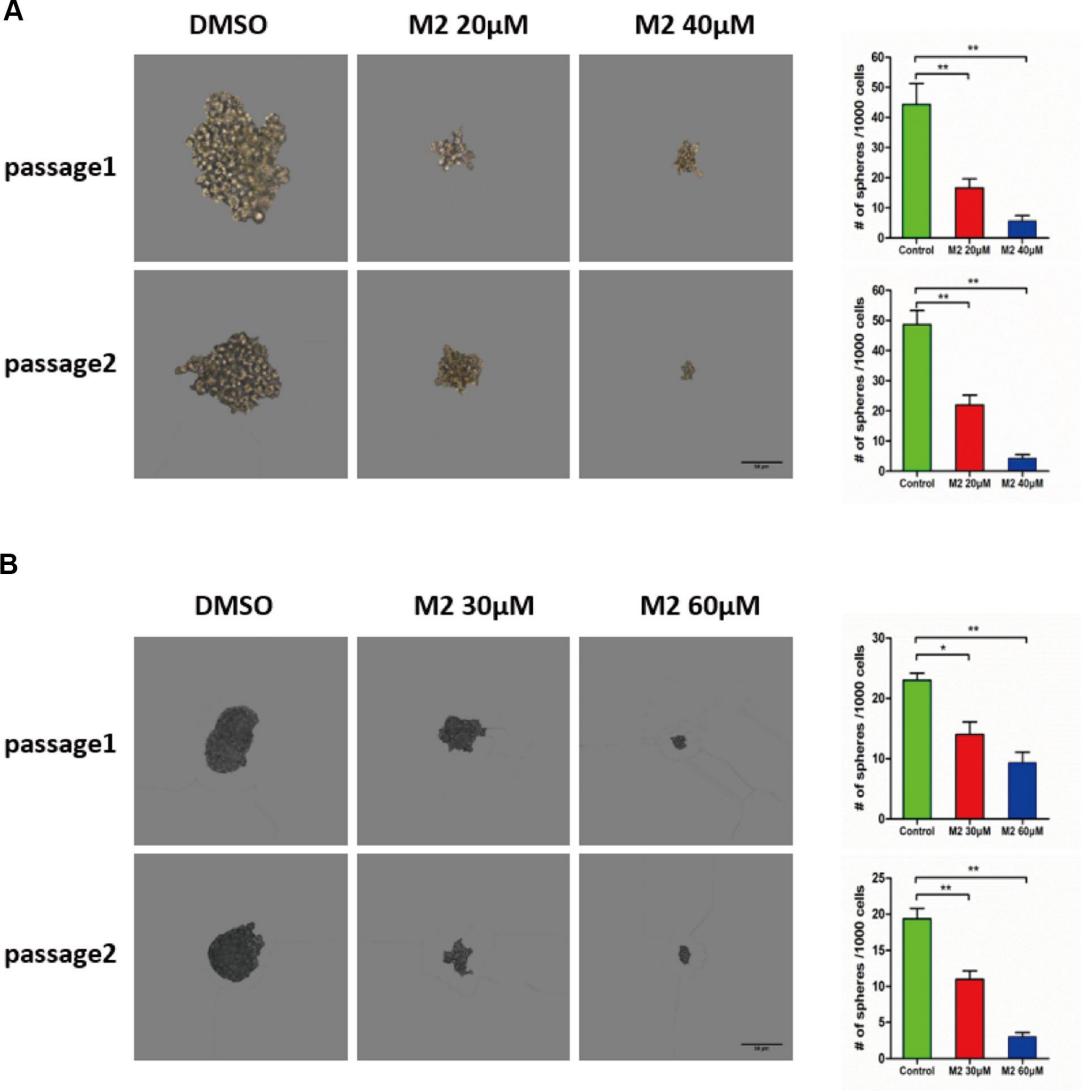
**Gomisin M2 suppresses CSC self-renewal.** (**A**) MDA-MB-231 cells were treated with 20 μM or 40 μM Gomisin M2 or DMSO control for 48 h. In the absence of Gomisin M2, the first and second passages that were derived from Gomisin M2-treated primary tumor spheres produced a smaller number of spheres compared with the DMSO control. (**B**) HCC1806 cells were treated with 30 μM or 60 μM Gomisin M2 or DMSO control for 48 h. The same method was applied to HCC1806 cells. Right panel, the number of two-passage spheres compared with the control. The data are presented as the mean ± SD. **, P<0.01.

Emerging evidence has demonstrated that cancer stem cells accumulate in non-adherent tumor spheres. To explore whether Gomisin M2 could inhibit the formation of tumor spheres *in vitro*, we treated the MDA-MB-231 CSC and HCC1806 CSC populations with Gomisin M2 for 48 h and performed two additional tumor sphere cultures without Gomisin M2. The results showed that the size and number of tumor spheres significantly decreased after Gomisin M2 treatment (Figure 3A and 3B).

### Mitochondria-initiated events are induced by Gomisin M2 and doxorubicin tumor spheres

**Figure 4:**
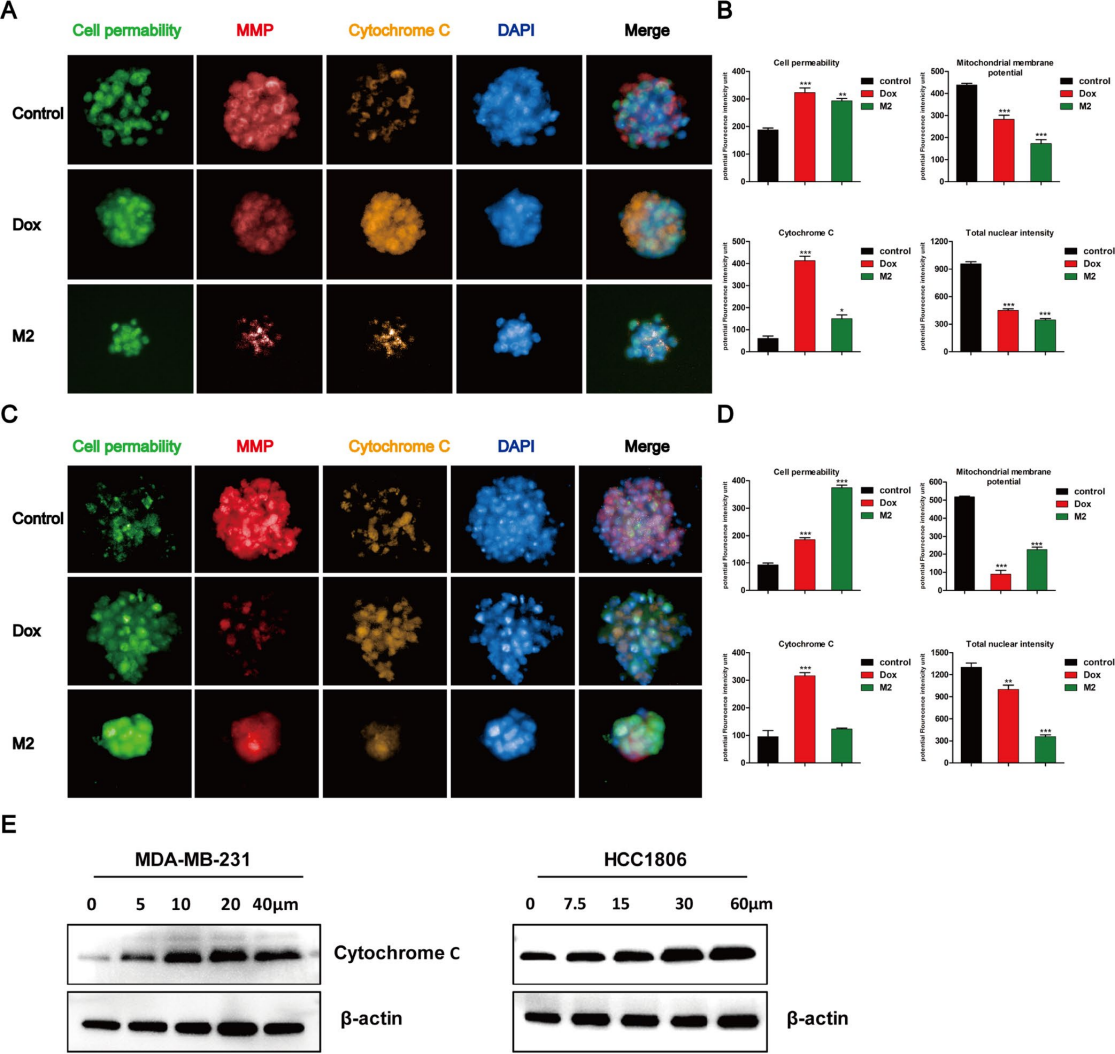
**Tumor spheres treated with Gomisin M2 for 48 h.** Treated MDA-MB-231 (**A**) and HCC1806 (**C**) tumor spheres were stained with cell permeability, MMP, cytochrome c, and DAPI dyes. Tumor spheres treated with 0.1% vehicle DMSO were used as negative controls. Doxorubicin was used as positive control. (**B**) The quantitative data of panel A (MDA-MB-231) tumor spheres. The flourecence intencity of cell permeability, MMP, cytochrome c and total nuclear are shown. (**D**) The quantitative data of panel C (HCC1806) tumor spheres. The flourecence intencity of cell permeability, MMP, cytochrome c and total nuclear are shown. ** p < 0.01, *** p < 0.0001, t-test. (**E**) Western blot analysis of cytosolic cytochrome c release in the cytosol of MDA-MB-231 and HCC1806 cell lines after 48 h of treatment, respectively.

Four independent parameters that monitor tumor spheres health, including nuclear size, mitochondrial membrane potential changes, cytochrome c release, and changes in cell permeability (Figure 4A and 4C). The DAPI dye enables monitoring of cell loss and DNA content, which is proportional to the total DAPI intensity per nucleus. The other three parameters are monitored by separate dyes. Gomisin M2 and doxorubicin were administered to the MDA-MB-231 and HCC1806 tumor spheres for 48 h and assessed using an ArrayScan High-Content Screening (HCS) system (Cellomics). The quantification results indicated that the flourescence intensity of cell permeability and cytochrome c increased, whereas MMP significantly decreased after 48 h of doxorubicin and Gomisin M2 treatment according to total intensity divided by volume. There was a decrease of blue fluorescence in total nuclear intensity after 48 h of doxorubicin and Gomisin M2 treatment (Figure 4B and 4D). In addition, the expression of cytochrome c significantly increased after the application of different concentrations of Gomisin M2 for 48 h both in MDA-MB-231 and HCC1806 (Figure 4E).

### Gomisin M2 induces apoptosis in breast cancer cells

**Figure 5:**
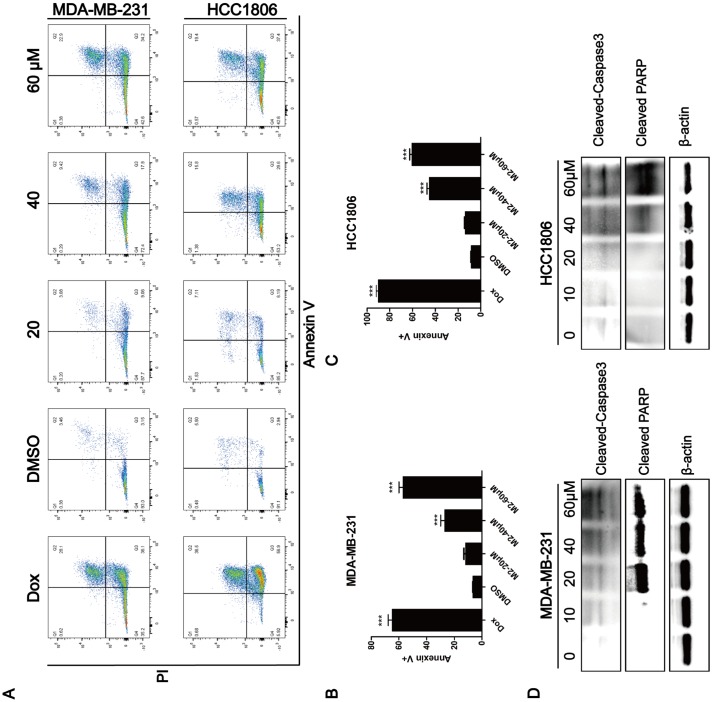
**Induction of apoptosis by Gomisin M2.** (**A**) After 48 h of incubation with Gomisin M2 at the indicated concentrations, cell apoptosis was assessed by flow cytometry after staining with propidium iodide (PI) and Annexin V. Doxorubicin was used as positive control. (**B**) The quantitative data of panel a (MDA-MB-231 cells). The percentages of Annexin V-positive cells are shown. ** p < 0.01. (**C**) The quantitative data of panel a (HCC1806 cells). (**D**) Western blot analysis of apoptotic hallmarks, treated with different doses of Gomisin M2 for 48 h.

The observation that Gomisin M2 significantly decreased cell viability within 48 h suggested that Gomisin mainly induces apoptosis in breast cancer cells. We analyzed apoptosis in Gomisin M2-treated breast cancer cells by flow cytometry. Cells were treated with 10 μM, 20 μM, 40 μM, and 80 μM of DMSO for 48 h. The results showed that Gomisin M2 significantly increased the number of apoptotic cells both in MDA-MB-231 and HCC1806 cell lines in a dose-dependent manner (Figure 5A). Then flow cytometry analysed the percentages of apoptosis in the MDA-MB-231 (Figure 5B) and HCC1806 cells (Figure 5C). Moreover, Gomisin M2 induced Cleaved caspase-3 and Cleaved PARP expression in both cell lines in a dose-dependent manner (Figure 5D).

### Monitoring the effects of Gomisin M2 on DNA replication in breast cancer cells by BrdU assay

**Figure 6:**
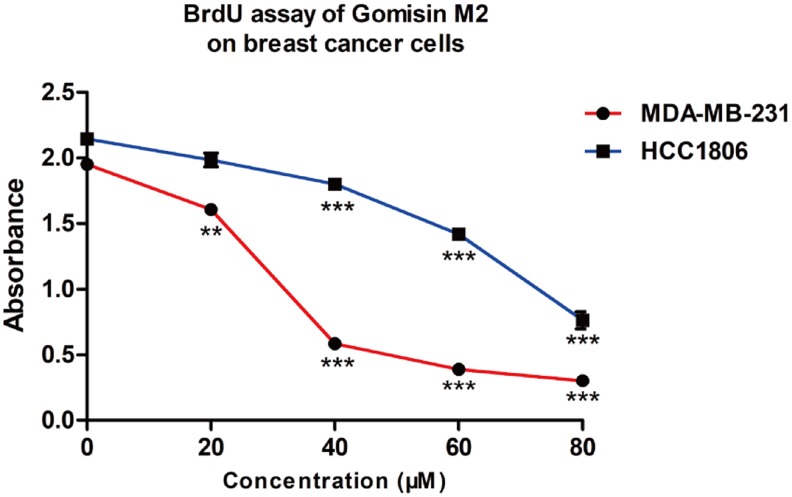
**The proliferative effect of Gomisin M2 on MDA-MB-231 and HCC1806 cells following 48 h treatment.** (Data are expressed as the mean ± SD relative to the vehicle control of three independent experiments ** p < 0.01, *** p < 0.0001).

To investigate whether Gomisin M2 induces cell proliferation inhibition by blocking the DNA synthesis, we evaluated the effect of Gomisin M2 on DNA replication in MDA-MB-231 and HCC1806. As shown in Figure 6, the results of BrdU (bromodeoxyuridine) assay demonstrated that Gomisin M2 had inhibitory effect on cell proliferation and DNA synthesis.

### Gomisin M2 downregulates the Wnt/β-Catenin pathway in breast cancer cells

To investigate the mechanisms involved in the effects of Gomisin M2 on breast CSCs, we assessed the involvement of key signal transduction pathways implicated in tumor progression by western blot analysis. Many studies have shown that the Wnt/β- catenin pathway plays a key role in regulating stem cell self-renewal. Our results clearly showed that Gomisin M2 significantly downregulated CyclinD1, β-catenin, or p-GSK3β, and upregulated p-β-catenin or GSK3-β in a dose- and time-dependent manner in MDA-MB-231 and HCC1806 cells.

### Gomisin M2 inhibits growth and proliferation of a CSC-enriched breast cancer cell xenograft zebrafish model

**Figure 7:**
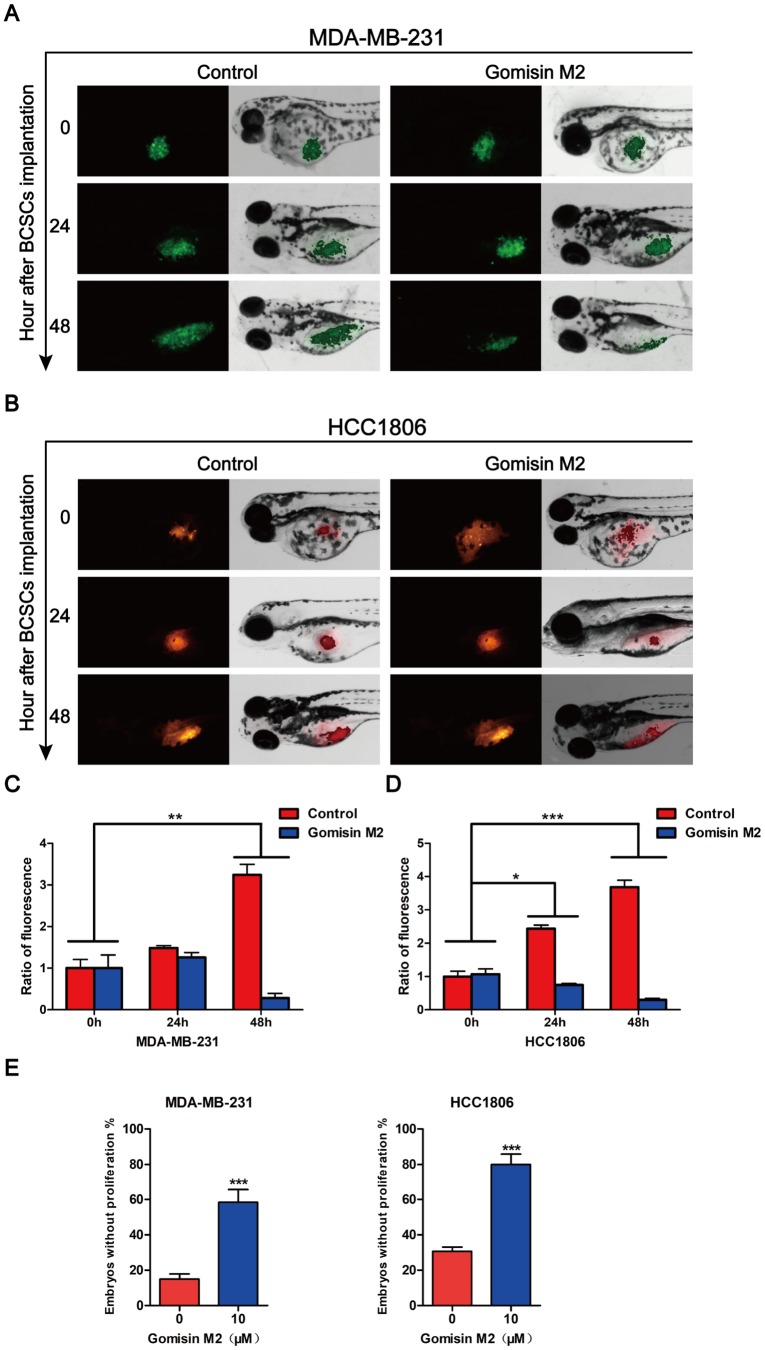
**Gomisin M2 inhibits tumor growth in zebrafish.** Cancer stem cells enriched MDA-MB-231-GFP (green) (**A**) or HCC1806 (red) (**B**) cells were microinjected into zebrafish embryos (larvae stage, n = 30 per group). Fluorescence density was captured by fluorescence microscopy at 0, 24 and 48 h after implantation. (**C**) The quantitative data of panel A in green fluorescence intensity. (**D**) The quantitative data of panel B in red fluorescence intensity. *p < 0.05, **p < 0.01, ***p < 0.001. (**E**) Statistical analysis of panel A and B the percentages of embryos without proliferation in control and Gomisin M2 groups in 48 h.

To further assess the anti-BCSC effect of Gomisin M2 *in vivo*, a zebrafish model was used for evaluation. CSC-enriched MDA-MB-231-GFP (Figure 7A) or HCC1806 (Figure 7B) cells labeled with DiI of 200-300 cells in PBS were microinjected into the 2-dpf zebrafish embryos. Then, the zebrafish embryos were treated with 10 μM Gomisin M2. Fluorescence density was captured by fluorescence microscopy at 0 h, 24 h and 48 h after implantation. Figure 7 illustrates that Gomisin M2 treatment dramatically reduced the growth and proliferation in CSC-enriched MDA-MB-231 or HCC1806 cells. The quantification results indicated that the flourecence intencity was gradually reduced in 48 h in the Gomisin M2 group compared with the control group (Figure 7C and 7D). In addition, the percentages of embryos without proliferation significantly increased in 48 h compared with DMSO group (Figure 7E).

## DISCUSSION

TNBC is a malignant subtype of breast cancer with poor prognosis [25]. A previous study showed that TNBCs are enriched for cancer stem cells [26]. Accumulating evidence has suggested that CSCs are related to tumor recurrence and drug resistance [27, 28]. Thus, targeting CSCs may provide a useful approach in controlling tumor resistance and lower relapse [29]. In this study, Gomisin M2 showed strong cytotoxicity against MDA-MB-231 and HCC1806 cancer lines (IC50 = 57~60μM). Furthermore, Gomisin M2 showed low cytotoxic activities against MCF10A non-cancer cells *in vitro* (Figure 1C). The compound of Gomisin M2 may be used as potential lead molecule against MDA-MB-231 and HCC1806 cancer cells. We demonstrated that Gomisin M2 significantly inhibits the BCSC proliferation and promotes apoptosis *in vitro* and *in vivo* zebrafish xenograft model. More importantly, Gomisin M2 induced apoptosis in TNBC cells via the PARP and Caspase-3 protein cleavage (Figure 5D).

To evaluate the effect of Gomisin M2 on BCSCs, we first analyzed the activity of Gomisin M2 against the breast cancer stem cells (with the CD44+/CD24- biomarkers) in MDA-MB-231 and HCC1806 cells. This is the first report that describes the *in vitro* effects of Gomisin M2, a natural compound isolated from the herb Schisandra viridis A. C. Smith, against both MDA-MB-231 and HCC1806 cells collected from MDA-MB-231 and HCC1806 stem cells/progenitors. We have demonstrated that Gomisin M2 (20~60μM) dramatically inhibited mammosphere evolution in both MDA-MB-231 and HCC1806 cells (Figure 3). BCSCs enriched in non-adherent round bunches of cells are called tumor spheres. We investigated whether breast CSCs after Gomisin M2 treatment was performed induced apoptosis by altering cell permeability, mitochondrial function, and caspase activities, thereby leading to loss of mitochondrial membrane potential and release of cytochrome c from the mitochondria. Based on their role in directly activating apoptosis programs in cells, mitochondria are considered as significant players in the course of apoptosis [30]. Then high-content system florescence analysis showed that Gomisin M2 targets the mitochondria, leading to loss of MMP, and later, causes apoptotic alterations in treated MDA-MB-231 and HCC1806 tumor spheres (Figure 4C and 4D). Although doxorubicin and Gomisin M2 significantly decreased the mitochondrial membrane potential flourecence intensity at the same concentration of 20 μM, doxorubicin group did not significantly reduce the size of the tumor sphere compared with the Gomisin M2 group (Figure 4A and 4B). The results indicate that Gomisin M2 had the effect of targeting derived MDA-MB-231 and HCC1806 CSCs. The activation of caspase is a key regulator of apoptosis [31–32]. The treated breast cancer cells exhibited a significant increase in Cleaved caspase-3 and Cleaved PARP expression 48 h after treatment (Figure 5D).

**Figure 8:**
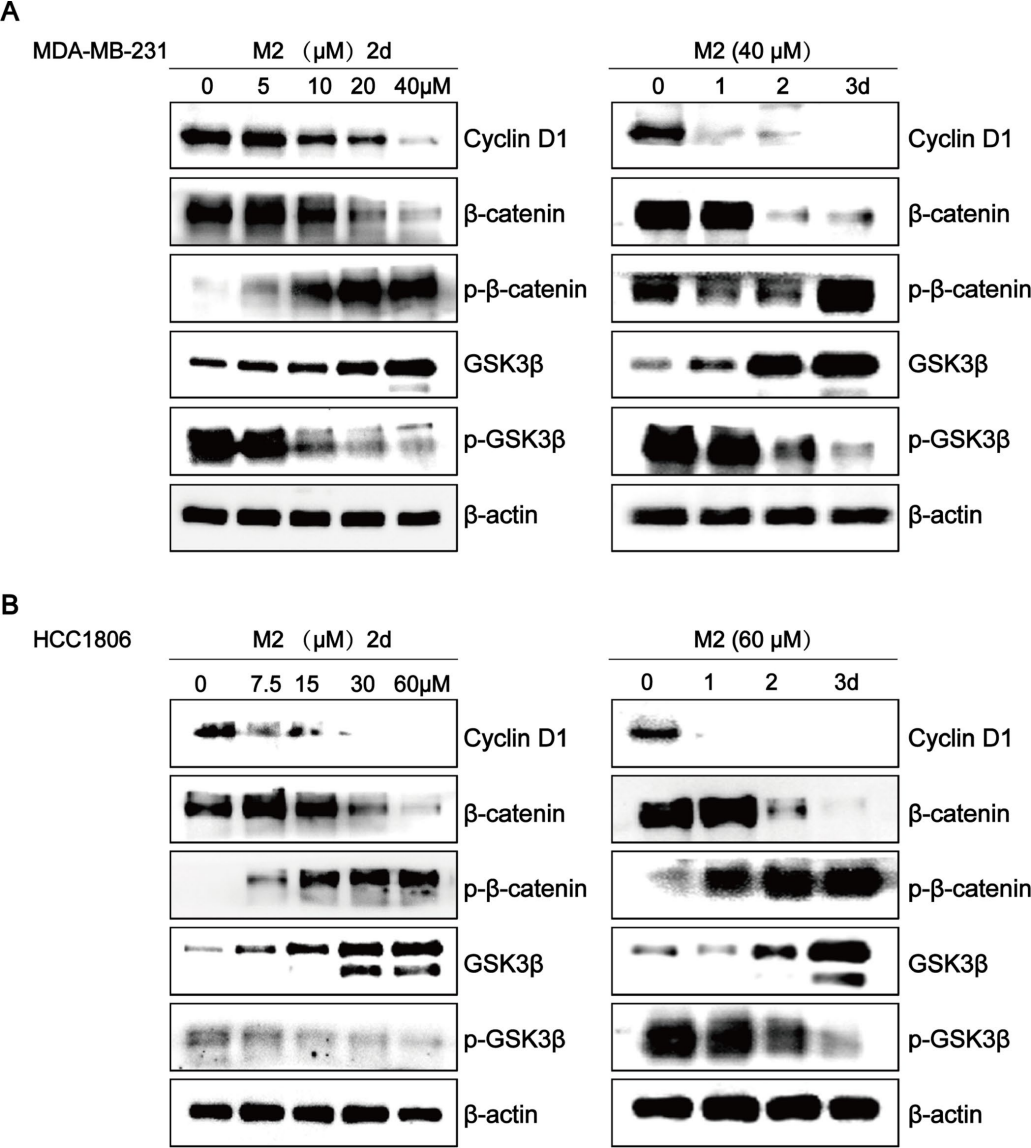
**Gomisin M2 downregulates the Wnt/β-Catenin pathway in breast cancer cells.** The effects of Gomisin M2 on expression levels of cyclin D1, β-catenin, GSK3-β, p-β-catenin, and p-GSK3β in MDA-MB-231 (**A**) and HCC1806 (**B**) cells. Cells were stimulated with increasing doses of Gomisin M2 for 48 h, and the same dose was applied at different time points. The experiment was repeated thrice with similar results.

Several reports have shown that Wnt/β-catenin and Hedgehog play key roles in self-renewal behavior of breast CSCs [32–34]. The Wnt/β-catenin pathway is involved in the regulation of cell proliferation, migration, apoptosis, differentiation, and stem cell self-renewal [35–37]. In our study, western blot analyses of MDA-MB-231 and HCC1806 breast cancer cells CSCs showed that Gomisin M2 downregulates the Wnt/β-catenin self-renewal pathway and cyclin D1 expression via GSK3β activation. The level of β-catenin remained low due to degradation in the absence of Wnt signaling. Therefore, Wnt signaling plays a key role in maintaining CSCs of various cancers. The downregulatory effect of Gomisin M2 on the Wnt/β-catenin pathway in breast cancer cells is shown in Figure 8.

Zebrafish have recently emerged as an ideal *in vivo* model for human diseases, including a variety of human cancer types. Due to its small size, strong fertility, and fast maturation time, it has become an important new cancer model that complements the functions traditionally implemented in mouse and cell culture systems [38, 39]. This study demonstrated that Gomisin M2 blocks breast cancer stem cell growth and proliferation in zebrafish embryos as shown in Figure 7A and 7B. The implanted cells proliferate in the zebrafish and the potential reduction of growth and metastasis is not due to simple regression of the tumor cells itself. It is probable that the Gomisin M2 inhibits the stemness of cancer stem cells. One interesting finding is that the dose of Gomisin M2 that blocked the cell growth *in vivo* was approximately two-fold lower than those suppressing tumor spheres formation. This finding is unexpected and indicates that Gomisin M2 effectively targets tumor cells in the *in vivo* tumor microenvironment with a lower concentration than *in vitro*. In Figure 2C, we confirmed that HCC1806 stem cells underwent tail migration on the third day after implantation, although we did not observe any fluorescent particles that had migrated to the tail after Gomisin M2 treatment. Although we digested the MDA-MB-231 tumor spheres into single cells and injected these into the zebrafish, these did not migrate until the sixth day, suggesting that CD44+CD24- is not a suitable marker for MDA-MB-231 stem cells.

The findings of this research provide insights on Gomisin M2 targeting BCSCs and inhibiting breast cancer stem cell metastasis *in vivo*. Furthermore, our results showed that the downregulation of the Wnt/β-catenin signal pathway via Gomisin M2 as one of the possible mechanisms for its efficacy. These data suggest that Gomisin M2 may be potentially used in breast cancer treatment. In addition, further research is needed to confirm the effects of Gomisin M2 on cancer stem-like cells of other cancer cell lines and primary carcinomas.

## MATERIALS AND METHODS

### Compounds and antibodies

The compound of Gomisin M2 was deposited at the Guangxi Key Laboratory of Efficacy Study on Chinese Materia Medica, Guangxi University of Chinese Medicine, Nanning, China. Gomisin M2 dissolved in dimethyl sulfoxide (DMSO) at a concentration of 100 mM. Baizuan were discussed in detail in a previously published article [24]. Total proteins (20-25 μg) were subjected to 12% SDS-PAGE. Antibodies for western blots are as follows: cytochrome c (11940), GSK-3β (5676), β-catenin (9582), cyclin D1 (2978), phospho-β-catenin (4176), phospho-GSK-3β (9327), Cleaved Caspase-3 (9661), Cleaved PARP (9185) and β-actin (4970) from Cell Signaling Technology.

### Cell culture

MCF10A, MDA-MB-23, and HCC1806 human breast cancer cells were acquired from ATCC (Manassas, VA, USA). MCF10A was cultured in DMEM/Ham’s F-1250/50 medium supplemented with 5% horse serum, 10 μg/mL insulin, 0.5 μg/mL hydrocortisone, 20 ng/mL epidermal growth factor, 0.1 μg/mL cholera enterotoxin, and 2 mM L-glutamine. The MDA-MB-231 cells were cultured in DMEM. The HCC1806 cells were maintained in RPMI-1640 medium. MDA-MB-231-GFP constructs were established at the Guangxi Key Laboratory of Efficacy Study on Chinese Materia Medica, Guangxi University of Chinese Medicine. All media were purchased from Gibco and supplemented with heat-inactivated 10% fetal bovine serum (FBS; Gibco) and 1% penicillin/streptomycin (P/S; Gibco). Cells were maintained at 37°C with 95% humidity and 5% CO2.

### Cell viability assays

Cells were seeded at a density of 1,500 cells/well in 384-well plates containing 50 μL of complete medium in six replicate wells. The cells were allowed to attach overnight before treating with the indicated dose of compounds isolated from Baizuan for 48 h. Subsequently, Alamar blue (Thermo Scientific) was added to the wells. After 24 h, the plate was read using a microplate reader. The proliferation of the MCF10A, MDA-MB-231, and HCC1806 cells was measured using an Alamar Blue assay kit according to the manufacturer’s protocol.

### Effects of Gomisin M2 on 3D spheroid formation in breast cancer cells

For the generation of 3D spheroids, the MDA-MB-231 and HCC1806 cells were harvested and seeded into ULA 96-well plates (Corning, Cat. No. 7007, USA) with complete medium at a density of 10,000 cells/well. The round well shape and ULA-coating prevent cells from adhering to the polystyrene plate and promote cellular interaction and form the small spherical microtissues. By 24 hours, cells have come together to form aggregated multicellular spheroids. Then these spheroids were treated with 100 μM Gomisin M2 for nine days. Spheroid formation and growth was assessed using the Operetta High-Content Imaging System to photograph all spheroids in each plate at each time-point. Approximately three replicate tumor spheroid samples were used for quantification.

### Isolation of breast cancer stem cells with magnetic-activated cell sorting (MACS)

The CD44+/CD24- cells were sorted from MDA-MB-231 and HCC1806 cell lines using magnetic separation (MagCellect™, RD SYSTEMS, Mckinley Place NE, Minneapolis, MN, USA; Human CD44+/CD24- Breast Cancer Stem Cells Isolation Kit) according to the manufacturer’s instructions. Briefly, the first step was CD24-negative cell selection. The undesired cells were retained on the walls of the tubes while the cells of interest remained in suspension. The desired cells were recovered by carefully harvesting the cell suspension by aspiration. The second step was CD44-positive cell selection. The cells of interest are retained on the walls of the tubes while the undesired cells remain in suspension. After carefully aspirating the cells in suspension, the tube was removed from the magnet to collect the desired cells. Thereafter, the sorted CD44+/CD24-cells were seeded in ultra-low attachment surface polystyrene six-well plates (Corning, Cat. No. 3471, USA) for amplification culture with stem cell conditioned medium.

### Mammosphere culture

The magnetic bead-sorted cells were seeded into ultra-low attachment surface polystyrene six-well plates (Corning, Cat. No. 3471, USA) and cultured as tumor spheres in serum-free medium (Stemcell, Cat. No. 5620) according to the manufacturer’s guidelines. The number of spheres was counted 7–14 days after plating. Different concentrations of Gomisin M2 were added to the primary cultures, while the following passages were cultured in the absence of Gomisin M2. After one week of culture, the number of tumor spheres was counted was under GELCOUNTTM.

### Immunofluorescence

Tumor spheres were fixed with 4% paraformaldehyde for 10 min and permeabilized by using 0.5% Triton X-100. Then cells were blocked with 5% bovine serum albumin (Sigma) for 15 min and incubated with anti-CD44 (NOVUS; 1:1000) antibody overnight at 4°C. The cells were washed thrice with PBST and incubated with Alexa 488 or Alexa 594-conjugated secondary antibodies (Invitrogen). Nuclei were counterstained using DAPI (Signaling, 1:2,000). Fluorescence images of tumor spheres were captured using the Opera® High-Content Screening System.

### Multiparameter cytotoxicity assay

The Thermo Scientific Cellomics Multiparameter Cytotoxicity 3 Kit enables simultaneous measurement of six orthogonal cell-health parameters: cell loss, nuclear morphology, DNA content, cell membrane permeability, mitochondrial membrane potential changes and cytochrome c localization, and release from mitochondria. Tumor spheres were plated in ULA 96-well plates (Corning, Cat. No. 3474, USA) and incubated with Gomisin M2 (40 μM) and doxorubicin (40 μM) for 48 h. For other detailed steps, please refer to the instructions (Thermo Scientific, Cellomics® Multiparameter Cytotoxicity 3 Kit). Fluorescence images of tumor spheres were taken by Opera® High-Content Screening System.

### Zebrafish maintenance

Zebrafish were maintained in a self-recirculating aquarium at an average temperature of 28°C with a 14-h light and 10-h dark cycle. Adult specimens were fed twice a day on a diet of Hikari micropellets (Kyorin) and brine shrimp. This study was approved by the institutional committee for animal welfare of the Guangxi University of Chinese Medicine. Zebrafish and embryos were maintained according to standard procedures. Zebrafish used in this study were purchased from China Zebrafish Resource Center (CZRC).

### Implantation of breast cancer cells in zebrafish embryos

We used the wild-type AB zebrafish embryos at 2-dpf to cell injection. The breast cancer cells MDA-MB-231-GFP and HCC1806 were labeled with DiI derived from non-adherent tumor spheres. Single cell suspensions were loaded into a pulled glass micropipette. The embryos were transferred into water containing 0.02% tricane (MS-222) 2 min before injection. Zebrafish embryos were subsequently placed onto a 2% agarose gel. Approximately, 200–300 non-CSC and CSC cells were respectively injected into the yolk sac area, connecting the microcapsule glass capillary needle under a stereo microscope (Leica Microsystems). Then, the embryos were exposed to different Gomisin M2 concentrations. The exposed embryos were maintained at 28°C for 1 h and then transferred in an incubator at 34°C for one week. The medium was changed every two days.

### Flow cytometry analysis of apoptosis

MDA-MB-231 and HCC1806 cells were seeded in 25 cm2 flasks and treated with 20, 40, and 80 μM of Gomisin M2 or 1/1000 dimethyl sulfoxide (DMSO) for 48 h. Cells were stained with Annexin V and propidium iodide (PI), according to the manufacturer’s protocol of the FITC Annexin V Apoptosis Detection kit I (BD Biosciences, San Diego, CA). Finally, the cells were analyzed by flow cytometry.

### Cell proliferation assay

MDA-MB-231 and HCC1806 cells were seeded in 96-well plates at 2,000 cells/well and treated with the same concentrations of Gomisin M2 as those used in the Alamar Blue assay for 48 h. Add BrdU (BrdU ELISA kit, Abnova) to the wells for 2 h. After discarding the culture medium, Fixing/Denaturing solution was added for 30 min at room temperature and was removed thoroughly. Anti-BrdU Monoclonal Antibody was added for 1 h at room temperature. The cells were washed and incubated for 1 h at room temperature with HRP conjugated Anti-Mouse IgG. Measure absorbance at 450 nm.
