# Correction for: Gomisin M2 from Baizuan suppresses breast cancer stem cell proliferation in a zebrafish xenograft model

**DOI:** 10.18632/aging.204872

**Published:** 2023-07-14

**Authors:** Yeguo Yang, Erwei Hao, Xianglong Pan, Dechao Tan, Zhengcai Du, Jinling Xie, Xiaotao Hou, Jiagang Deng, Kun Wei

**Affiliations:** 1 School of Biology and Biological Engineering, South China University of Technology, Guangzhou 510006, China; 2 Guangxi Key Laboratory of Efficacy Study on Chinese Materia Medica, Guangxi University of Chinese Medicine, Nanning, Guangxi 530200, China; 3 Sino-Canada Joint Zebrafish Lab for Chinese Herbal Drug Screening, Guangxi University of Chinese Medicine, Nanning, Guangxi 530200, China; 4 Guangxi Collaborative Innovation Center for Research on Functional Ingredients of Agricultural Residues, Guangxi University of Chinese Medicine, Nanning, Guangxi 530200, China

**Keywords:** Gomisin M2, breast cancer stem cell, zebrafish xenografts, proliferation

**This article has been corrected:** The authors found errors in **Figure 7A** and **7B**. In **Figure 7A**, the fluorescence microscopy images of control and Gomisin M2-treated zebrafish embryos microinjected with cancer stem cell (CSC)-enriched MDA-MB-231-GFP (green) cells at 0 h after implantation were duplications of **Figure 2C** images of zebrafish embryos injected with non-CSC and CSC-enriched MDA-MB-231-GFP (green) cells at 0 time point. In **Figure 7B**, the fluorescence images of zebrafish embryos microinjected with CSC-enriched HCC-1806 cells (red) and treated with Gomisin M2 for 24 or 48 hours were a duplication of images of untreated control embryos at the same time points. The authors corrected all errors with the representative images from the original experiments. The presented corrections do not affect the results or conclusions of this article. The authors would like to apologize for any inconvenience caused.

Corrected **Figure 7** is presented below.

**Figure 7:**
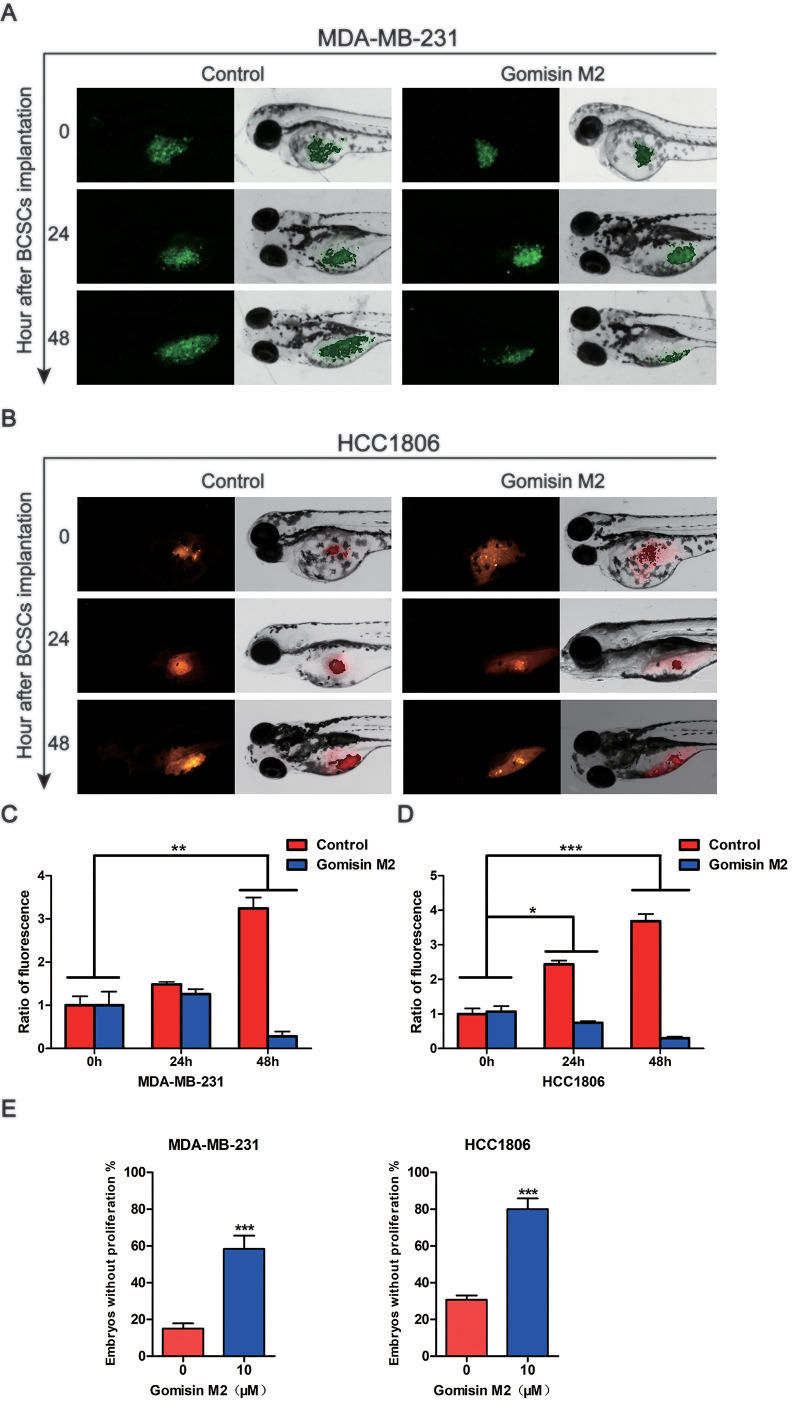
**Gomisin M2 inhibits tumor growth in zebrafish. **Cancer stem cells enriched MDA-MB-231-GFP (green) (**A**) or HCC1806 (red) (**B**) cells were microinjected into zebrafish embryos (larvae stage, n = 30 per group). Fluorescence density was captured by fluorescence microscopy at 0, 24 and 48 h after implantation. (**C**) The quantitative data of panel A in green fluorescence intensity. (**D**) The quantitative data of panel B in red fluorescence intensity. *p < 0.05, **p < 0.01, ***p < 0.001. (**E**) Statistical analysis of panel A and B the percentages of embryos without proliferation in control and Gomisin M2 groups in 48 h.

